# First experiences with machine learning predictions of accelerated declining eGFR slope of living kidney donors 3 years after donation

**DOI:** 10.1007/s40620-024-01967-y

**Published:** 2024-06-05

**Authors:** Leandra Lukomski, Juan Pisula, Tristan Wagner, Andrii Sabov, Nils Große Hokamp, Katarzyna Bozek, Felix Popp, Martin Kann, Christine Kurschat, Jan Ulrich Becker, Christiane Bruns, Michael Thomas, Dirk Stippel

**Affiliations:** 1 Department of General, Visceral, Cancer and Transplant Surgery, Faculty of Medicine and University Hospital of Cologne, Kerpener Straße 62, 50937 Cologne, Germany; 2 Data Science of Bioimages Lab, Center for Molecular Medicine Cologne (CMMC), Faculty of Medicine and University Hospital of Cologne, University of Cologne, Robert-Koch-Straße 21, 50937 Cologne, Germany; 3 Institute for Diagnostics and Interventional Radiology, Faculty of Medicine and University Hospital of Cologne, Kerpener Straße 62, 50937 Cologne, Germany; 4 Department II of Internal Medicine and Center for Molecular Medicine Cologne, Faculty of Medicine and University Hospital of Cologne, Kerpener Straße 62, 50937 Cologne, Germany; 5 Institute of Pathology, Faculty of Medicine and University Hospital of Cologne, Kerpener Straße 62, 50937 Cologne, Germany

**Keywords:** Living kidney donors, Machine learning in transplantation, eGFR slope, Living kidney transplantation

## Abstract

**Background:**

Living kidney donors are screened pre-donation to estimate the risk of end-stage kidney disease (ESKD). We evaluate Machine Learning (ML) to predict the progression of kidney function deterioration over time using the estimated GFR (eGFR) slope as the target variable.

**Methods:**

We included 238 living kidney donors who underwent donor nephrectomy. We divided the dataset based on the eGFR slope in the third follow-up year, resulting in 185 donors with an *average eGFR slope* and 53 donors with an *accelerated declining eGFR-slope*. We trained three Machine Learning-models (Random Forest [RF], Extreme Gradient Boosting [XG], Support Vector Machine [SVM]) and Logistic Regression (LR) for predictions. Predefined data subsets served for training to explore whether parameters of an ESKD risk score alone suffice or additional clinical and time-zero biopsy parameters enhance predictions. Machine learning-driven feature selection identified the best predictive parameters.

**Results:**

None of the four models classified the eGFR slope with an AUC greater than 0.6 or an *F*1 score surpassing 0.41 despite training on different data subsets. Following machine learning-driven feature selection and subsequent retraining on these selected features, random forest and extreme gradient boosting outperformed other models, achieving an AUC of 0.66 and an *F*1 score of 0.44. After feature selection, two predictive donor attributes consistently appeared in all models: smoking-related features and glomerulitis of the Banff Lesion Score.

**Conclusions:**

Training machine learning-models with distinct predefined data subsets yielded unsatisfactory results. However, the efficacy of random forest and extreme gradient boosting improved when trained exclusively with machine learning-driven selected features, suggesting that the quality, rather than the quantity, of features is crucial for machine learning-model performance. This study offers insights into the application of emerging machine learning-techniques for the screening of living kidney donors.

**Graphical abstract:**

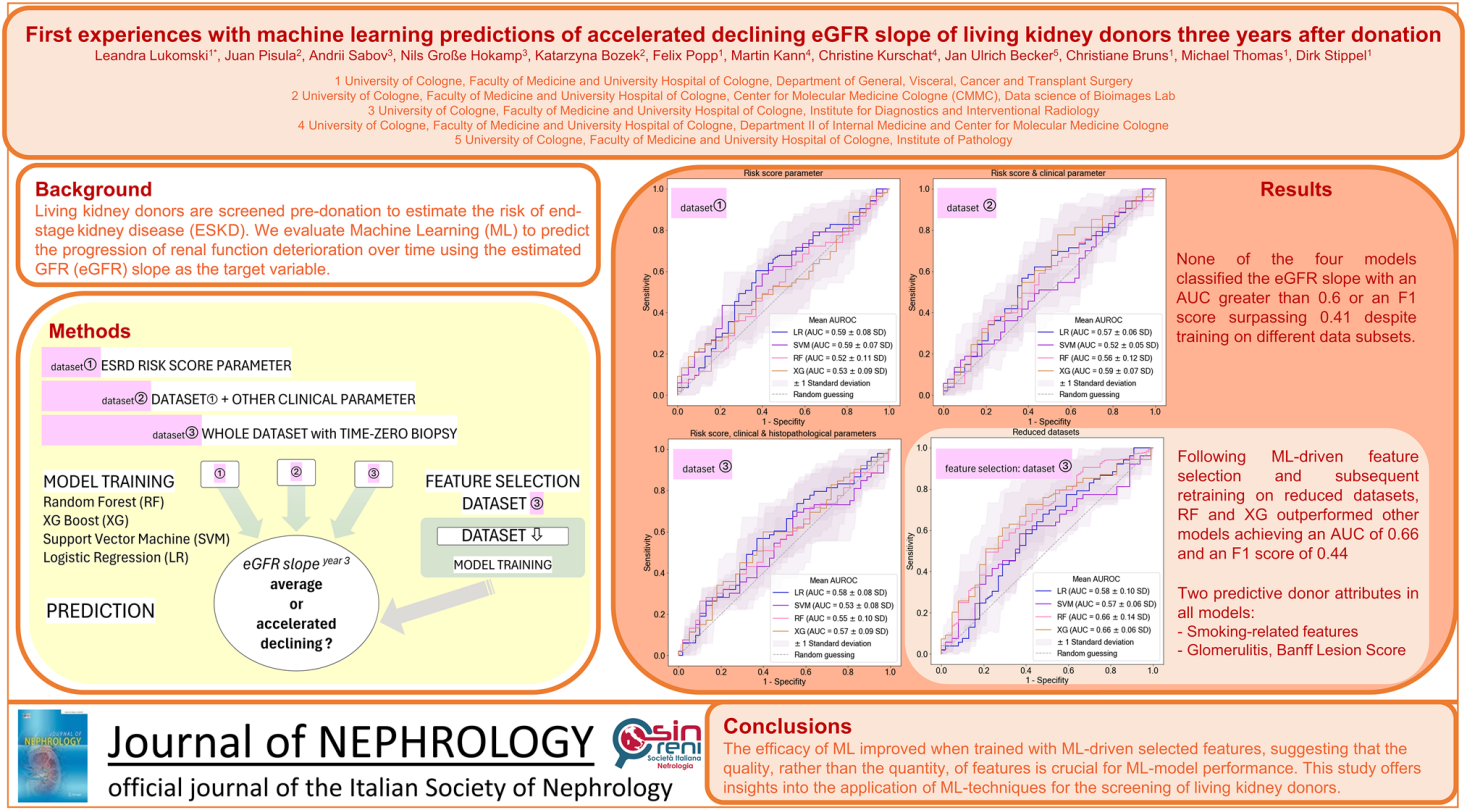

**Supplementary Information:**

The online version contains supplementary material available at 10.1007/s40620-024-01967-y.

## Introduction

Living kidney donors face the same risk of developing end-stage kidney disease (ESKD) as the general population [1, 2]. However, recent studies have called this statement into question [3, 4]. Many transplantation centers encounter a heterogeneous donor pool that is different from the healthy study cohorts of older investigations. Due to long transplantation waiting lists, donors with a lower starting glomerular filtration rate (GFR) or other risk factors such as smoking history may be eligible for donation.

Therefore, thorough screening before donation is essential. Various pre-donation risk assessments have been developed to identify the donors at risk for ESKD [5–7]. We use the ESKD risk score for donors which was first published in 2016 by Grams et al. [7]. All risk scores provide applicable tools for clinical practice but are based on statistical approaches. This is where Artificial Intelligence (AI) comes into play based on our hypothesis that artificial intelligence has the potential to improve predictions.

Whereas classic statistics outline relationships between a data sample and a population, Machine Learning (ML), a subgroup of artificial intelligence, is capable of making personalized predictions about a desired outcome by attempting to uncover hidden patterns within the provided data [8]. The goal of identifying borderline donors may be facilitated with machine learning, enabling this donor group to be educated in detail about their possibly increased risk of kidney failure after donation and initiating intensified follow-up care.

The main focus of machine learning studies in transplantation has been on the outcome of graft function and the prediction of graft failure [9–11]. When it comes to donors, machine learning research is very scarce. To our knowledge, there is only one recent work using machine learning, carried out by a Korean study group, to predict renal adaptation of living kidney donors [12].

Our study aims to test different machine learning techniques to classify the *average eGFR slope* or the *accelerated declining eGFR slope* of living kidney donors, utilizing distinct subsets of the provided data, including parameters from the ESKD risk score, clinical data, and histopathological parameters. We chose the eGFR slope as our target for predictions since it represents a dynamic parameter over time of kidney function.

## Methods

### Objects and inclusion criteria

For this retrospective study, a total of 238 living kidney donors (sex at birth, female/male [%]: 154 [65]/84 (35); mean age [standard deviation, SD]: 54 [10]) after donor nephrectomy between 2009 and 2020 at the Department of General, Visceral, Cancer and Transplant Surgery, University Hospital of Cologne, Germany, were included. Hand-assisted retroperitoneoscopic donor nephrectomy (HARP) was the surgical technique used [13]. Inclusion criteria were donors who had completed 3 years of postoperative follow-up with complete documentation of serum creatinine values pre-donation and at year 1, 2 and 3 after donation to calculate the estimated GFR (eGFR) at each time point. Included patient characteristics can be divided into three groups:

1. Clinical characteristics of the risk tool for ESKD for kidney donor candidates (age, sex at birth, eGFR, systolic blood pressure, hypertension medication, body mass index [BMI], urine albumin creatinine ratio [ACR] and smoking history) [7]. Non-insulin-independent diabetes and race were excluded from the dataset due to one-dimensional distribution. We excluded outliers (*n* = 2) in albumin creatinine ratio to ensure no distorted model performance.
2. Other donor characteristics assessed preoperatively (height, weight, smoking pack years, serum creatinine, side of the removed kidney, renal cortex volumetry of the graft and of the remaining kidney, and their ratio [remaining to transplant cortex volumetry]). Renal cortex volumetry was assessed from preoperative computed tomography (CT) scans [14].
3. Histopathological assessment of the time-zero biopsy of the graft (total glomeruli, global glomerulosclerosis, ratio glomerulosclerosis [global glomerulosclerosis to total glomeruli], Banff Lesion Scores [15] of glomerulitis *g*, tubular atrophy *ct*, and arteriolar hyalinosis *ah*). We omitted the other Banff Lesion Scores due to one-dimensional distribution. To ensure that only representative core biopsies were included, a minimum set of ten glomeruli was defined to be representative [16].

The final dataset comprised 22 donor features and a missing feature rate of 17.7%, mainly due to incomplete documentation of the time-zero biopsy. A detailed description of the feature distribution is provided in Table 1. The Ethics Committee of the Faculty of Medicine, University of Cologne, Germany, approved this retrospective study (reference number: 23-1462-retro) and waived the need for patient consent. Data analysis was performed in accordance with relevant guidelines, as outlined by the Transparent Reporting of a multivariable prediction model for Individual Prognosis or Diagnosis (TRIPOD) statement [17].

**Table 1:** Patient characteristics and correlation to eGFR slope with a cut-off decline of -1 mL/min/1.73 m^2^/year

		All *N* = 238	Average eGFR slope *n* = 185	Accelerated declining eGFR slope *n* = 53	*p*
eGFR slope (ml/min/1.73 m^2^/year): year 3 post-donation	*n*/median (IQR)	238/− 0.33 (− 0.6, 3.05)	185/1.93 (− 0.4, 3.57)	53/− 3.95 (− 4.88, − 3.02)	–
eGFR (ml/min/1.73 m^2^): year 1 post-donation	*n*/mean (SD)	238/60.67 (13.88)	185/58.86 (13.01)	53/66.99 (15.05)	**< 0.001**^**†**^
eGFR (ml/min/1.73 m^2^): year 2 post-donation	*n*/mean (SD)	238/61.35 (13.2)	185/61.07 (13.3)	53/62.31 (12.9)	0.55^†^
eGFR (ml/min/1.73 m^2^): year 3 post-donation	*n*/mean (SD)	238/61.75 (13.01)	185/62.98 (13.37)	53/57.45 (10.75)	**0.006**^**†**^
Donor risk score (%): 15-year incidence for ESKD	*n*/median (IQR)	225/0.11 (0.07, 0.16)	177/0.11 (0.08, 0.16)	48/0.08 (0.05, 0.16)	0.04*
Donor risk score (%): lifetime incidence for ESKD	*n*/median (IQR)	225/0.31 (0.2, 0.51)	177/0.31 (0.2, 0.52)	48/0.31 (0.2, 0.49)	0.79*
**ESKD donor risk score parameters**					
Age at donation (years)	*n*/mean (SD)	238/54 (10)	185/54 (10)	53/51 (11)	0.06^†^
Sex at birth: female/male	Frequency (%)	154 (65)/84 (35)	117 (63)/68 (37)	37 (70)/16 (30)	0.47^‡^
BMI (kg/m^2^)	*n*/mean (SD)	228/26.9 (4.4)	185/26.9 (4.4)	53/26.7 (4.4)	0.71^†^
Systolic blood pressure (mmHg)	*n*/mean (SD)	233/129 (14)	182/129 (14)	51/129 (12)	0.95^†^
Hypertension medication: no/yes	Frequency (%)	170 (71)/68 (29)	134 (72)/51 (28)	36 (68)/17 (32)	0.64^‡^
Smoking history: non/former/current	Frequency (%)	162 (68)/39 (16)/37 (16)	120 (65)/33 (18)/32 (17)	42 (79)/7 (13)/4 (8)	0.11^‡^
eGFR (ml/min/1.73 m^2^): pre-donation	*n*/mean (SD)	238/92.67 (13.34)	185/91.9 (13.42)	53/95.37 (12.81)	0.09^†^
Urine albumin creatinine ratio (mg/g): pre-donation	*n*/median (IQR)	230/3 (0, 6)	179/3 (0, 6)	51/3 (0, 6)	0.66*
**Other clinical parameters**					
Body weight (kg)	*n*/mean (SD)	238/78 (15)	185/78 (15)	53/77 (14)	0.61^†^
Body height (cm)	*n*/mean (SD)	238/170 (9)	185/170 (9)	53/170 (9)	0.83^†^
Pack years (packs of cigarettes/year)	*n*/median (IQR)	201/0 (0, 0)	152/0 (0, 0)	49/0 (0, 0)	0.35*
Serum creatinine pre-donation	*n*/median (IQR)	238/0.8 (0.7, 0.9)	185/0.8 (0.7, 0.9)	53/0.8 (0.7, 0.9)	0.49*
Side of removed kidney: left/right	Frequency (%)	157 (66)/81 (34)	126 (68) /59 (32)	31 (58) /22 (42)	0.25^‡^
Renal cortex volumetry (cc): remaining kidney	*n*/mean (SD)	185/87.88 (22.49)	144/88.88 (23.37)	41/84.02 (18.82)	0.22^†^
Renal cortex volumetry (cc): graft	*n*/mean (SD)	185/86.35 (23.81)	144/88.03 (24.77)	41/80.44 (19.16)	0.25^†^
Ratio cortex volumetry	*n*/median (IQR)	185/1 (0.9, 1.2)	144/1 (0.9, 1.1)	41/1 (1, 1.2)	0.22*
**Histopathological parameters**					
Banff g: g0/g1/g2	Frequency (%)	102 (43)/13 (5)/2 (1)	81 (44)/10 (5)/1 (1)	21 (40)/3 (6)/1 (1)	0.47^¬^
Banff ct: ct0/ct1	Frequency (%)	108 (45)/7 (3)	86 (46)/5 (3)	22 (42)/2 (4)	0.63^¬^
Banff ah: ah0/ah1	Frequency (%)	90 (38)/27 (11)	69 (37)/23 (12)	21 (40)/4 (8)	0.42^¬^
Total glomeruli (no.)	*n*/median (IQR)	120/23 (16, 31)	97/20 (15, 31)	23/25 (20, 30)	0.27*
Global glomerulosclerosis (no.)	*n*/median (IQR)	120/0 (0, 1)	97/0 (0, 1)	23/1 (0, 2)	0.23*
Ratio glomerulosclerosis	*n*/median (IQR)	120/0 (0, 0.1)	97/0 (0, 0.1)	23/0 (0, 0.1)	0.54*

*eGFR* estimated glomerular filtration rate, *ESKD* end-stage kidney disease, *g* glomerulitis, *ct* tubular atrophy, *ah* arteriolar hyalinosis, *No.* number

^†^*t*-test, *Whitney-*U* test, ^‡^χ^2^ test, ^¬^Fisher exact test

### Labeling, feature pre-processing and engineering

The dataset was dichotomized into two groups based on the overall decline in eGFR (eGFR slope) over the first, second, and third year after donation. We defined an *average* decline of the eGFR in year 3 of the follow-up at a rate of < 1 mL/min/1.73 m^2^/year (*average eGFR slope*) based on the normal decline in kidney function of approximately 1 mL/min/1.73 m^2^/year [18]. An *accelerated decline* of the eGFR in year 3 at a rate of ≥ 1 mL/min/1.73 m^2^/year was considered a relevant deterioration in kidney function and is referred to as an *accelerated declining eGFR slope* throughout the remainder of this study for easier readability. Labeling resulted in an unbalanced dataset (*average eGFR slope*: 185 donors, 78%; *accelerated declining eGFR slope*: 53 donors, 22%). We used class weights in favor of the underrepresented class. We performed feature engineering of the 7 categorical and 15 continuous variables within *scikit learn* Pipelines to ensure proper pre-processing of the respective training and test data. We normalized continuous variables to impute missing data points using *scikit learns’s* *k*-Nearest Neighbor imputer (*n_neighbors* = 3). Missing values in categorial data were imputed with the most frequent variable. All categorical features were then converted into dummy variables with one-hot-encoding. In case of binary variables, the first dummy variable was dropped.

### Feature selection via sequential forward selection

We performed machine learning-driven sequential forward selection (SFS) for each algorithm on the entire dataset using the open-source *MLxtend* library [19]. This methodology is considered model-agnostic, meaning that feature selection is independent of the architecture of the model but is based on its influence on performance metrics [20]. The best estimator of each model after hyperparameter search was utilized for sequential forward selection with stratified 5 Cross-Validation (CV)-folds aiming to find the smallest subset of features for the best cross-validation-model performance. The evaluation of model performance after feature selection on the training folds was conducted solely on the respective testing fold to prevent data leakage. The important features that were identified served as a reduced dataset for model training, respectively.

### Study design

**Fig. 1:**
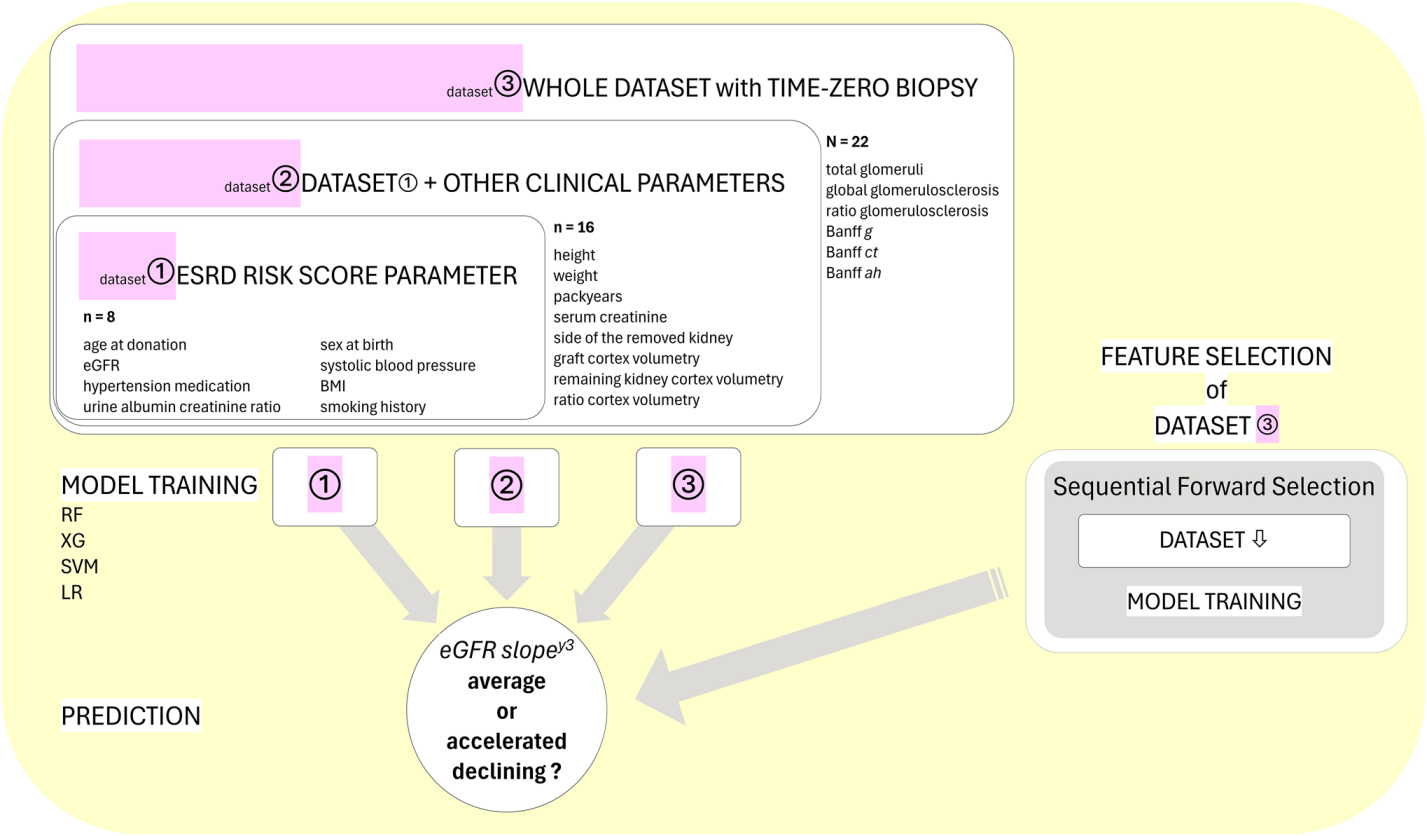
Flow diagram of the study design. First, distinct subsets (dataset 1 and 2) and the whole dataset 3 were used for model training with Random Forest (RF), XG Boost (XG), Support Vector Machine (SVM) and Logistic regression (LR) to classify eGFR slope of living kidney donors in the third follow-up year (y3). Second, for each model, ML-driven feature selection was performed on the entire dataset resulting in a correspondingly selected feature dataset for model retraining and predictions

The study design contains two major parts to classify the eGFR slope at year three post-donation (Fig. 1):

1. We utilized both the entire dataset and two predefined subsets generated from the entire dataset for model training to evaluate model performance:
  *Dataset 1*: Parameters of the ESKD risk score (*n* features = 8, *n* features after one-hot encoding = 10)
  *Dataset 2*: Dataset 1 + other clinical parameters (*n* features = 16, *n* features after one-hot encoding = 18)
  *Dataset 3*: Whole dataset including histopathological parameters (*N* features = 22, *N* features after one-hot encoding = 26)
2. Feature Selection with sequential forward selection was only performed on Dataset 3 for each model. We subsequently utilized the selected important features to retrain the models and to evaluate model performance, respectively.

### Machine learning models

We used supervised machine learning techniques for binary classification using the *scikit learn* package [21] unless specified otherwise.

*Random Forests (RF)* depend on multiple decision trees to finally predict the target. Random forests rely on bootstrap aggregation, called *bagging,* which implies the creation of subsets of training features (decision trees) to enhance model performance [22].

*Extreme Gradient Boosting (XG-Boost, XG)* as a further decision tree-based ensemble method proceeds, in addition to *bagging*, with the sequential highlighting of correctly classified subsamples for further predictions, called *boosting*. We used the extreme gradient boosting library in this study [23].

*Support Vector Machines (SVM)* create a decision boundary in a space*, a hyperplane*, to correctly classify the input features. The best hyperplane maximizes the distance to the nearest element of each target [24].

*Logistic Regression (LR)* uses the logistic function for dichotomous classification [25], and served as a state-of-the-art benchmark for the performance of the machine learning models used in this study. We used L1 (*Lasso*) regularization in all logistic regression models.

### Statistical analysis

The eGFR was calculated using the Chronic Kidney Disease Epidemiology Collaboration (CKD-EPI 2009) equation [26]. The eGFR slope was calculated by using the following formula: ${\text{eGFRslope}}=\frac{\Delta {\text{eGFR}}}{\Delta t}$, where $\Delta eGFR$ is the change in eGFR over the first-, second-, and third-year post-donation and $\Delta t$ is the time interval between eGFR calculations in years. We evaluated model performance with nested-cross-validation due to small data size. Briefly, the dataset is passed through two cross-validation loops to find optimal hyperparameters (inner loop) and to evaluate model performance after hyperparameter tuning (outer loop). We defined a range of hyperparameters (Supplementary Table 1) for each model and narrowed it down by empirical testing. We set the inner cross-validation loop to stratified 3 folds and the outer cross-validation loop to stratified 5 folds. The model performance is displayed by the *k*-fold *F*1 score (mean of the fivefold *F*1 scores) with its mean SD and the cross-validation receiver operating characteristic (ROC) curves (mean of the fivefold ROCs) with its corresponding *k*-fold area under the curve (mean AUC). *F*1 score is the best metric for class imbalance and is defined by the harmonic mean of precision and sensitivity [27]. We performed data analysis with Python (version 3.8.8) using the open source packages *pandas* (version 1.4.3) [28], *NumPy* (version 1.21.5) [29], *matplotlib* (version 3.5.1) [30], *seaborn* (version 0.11.2) [31], *missingno* (version 0.5.1) [32], and *scikit learn* (version 1.0.2) [21]. Statistical analysis was performed with SciPy (version 1.7.3) [33] and RStudio (version 1.1.456) [34]. Normal distribution of continuous variables was tested by Kolmogorov–Smirnov test. Continuous variables with normal distribution are presented as mean (SD), whereas non-normal variables are reported as median (interquartile range [IQR]). Categorical features are presented as frequencies (percentage). Independent samples Student’s *t* test was used to compare stratified, normally distributed continuous features. Mann–Whitney *U* test was used to compare skewed continuous variables. For comparison of the frequencies of categorical variables, *χ*^2^ or Fisher’s exact test were used. Parametric and non-parametric tests were performed two-sided. A value of *p* < 0.05 was considered significant.

## Results

### Baseline characteristics of donors and descriptive statistics

Across all inspected donors fulfilling the 3-year follow-up, the median eGFR slope (IQR) was -0.33 mL/min/1.73 m^2^/year (− 0.6, 3.05). In year three post-donation, 185 donors exhibited an *average eGFR slope* (median eGFR slope [IQR]: 1.93 mL/min/1.73 m^2^/year [− 0.4, 3.57]), and 53 donors revealed an *accelerated declining eGFR slope* (median eGFR slope [IQR]: − 3.95 mL/min/1.7 3m^2^/year [− 4.88, − 3.02]). In the *average eGFR slope* cohort, the mean age at donation (SD) was 54 years (10), 63% were female at birth, the median pre-donation ESKD risk score (IQR) was 0.11% (0.08, 0.16) for the 15-year and 0.31% (0.2, 0.52) for the lifetime incidence of ESKD, the mean eGFR (SD) was 58.86 mL/min/1.73 m^2^ (13.01) in the first year, 61.07 mL/min/1.73 m^2^ (13.3) in the second year, and 62.98 mL/min/1.73 m^2^ (13.37) in the third year after donation (Supplementary Fig. 1). In the *accelerated declining eGFR slope* cohort, the mean age at donation (SD) was 51 years (11), 70% were female at birth, the median pre-donation ESKD risk score (IQR) was 0.08% (0.05, 0.16) for the 15-year and 0.31% (0.2, 0.49) for the lifetime incidence of ESKD, the mean eGFR (SD) was 66.99 mL/min/1.73 m^2^ (15.05) in the first year, 62.31 mL/min/1.73 m^2^ (12.9) in the second year, and 57.45 mL/min/1.73m^2^ (10.75) in the third year after donation (Supplementary Fig. 1). A statistically significant difference between the two cohorts was found for eGFR in the 1- and 3-year follow-up, as well as for the donor ESKD risk score for the 15-year incidence of ESKD. However, these parameters were not included for model training. None of the parameters used for model training revealed any statistically significant difference between the two cohorts (*p* > 0.05) (Table 1).

### Machine learning predictions of accelerated declining eGFR slope

**Fig. 2:**
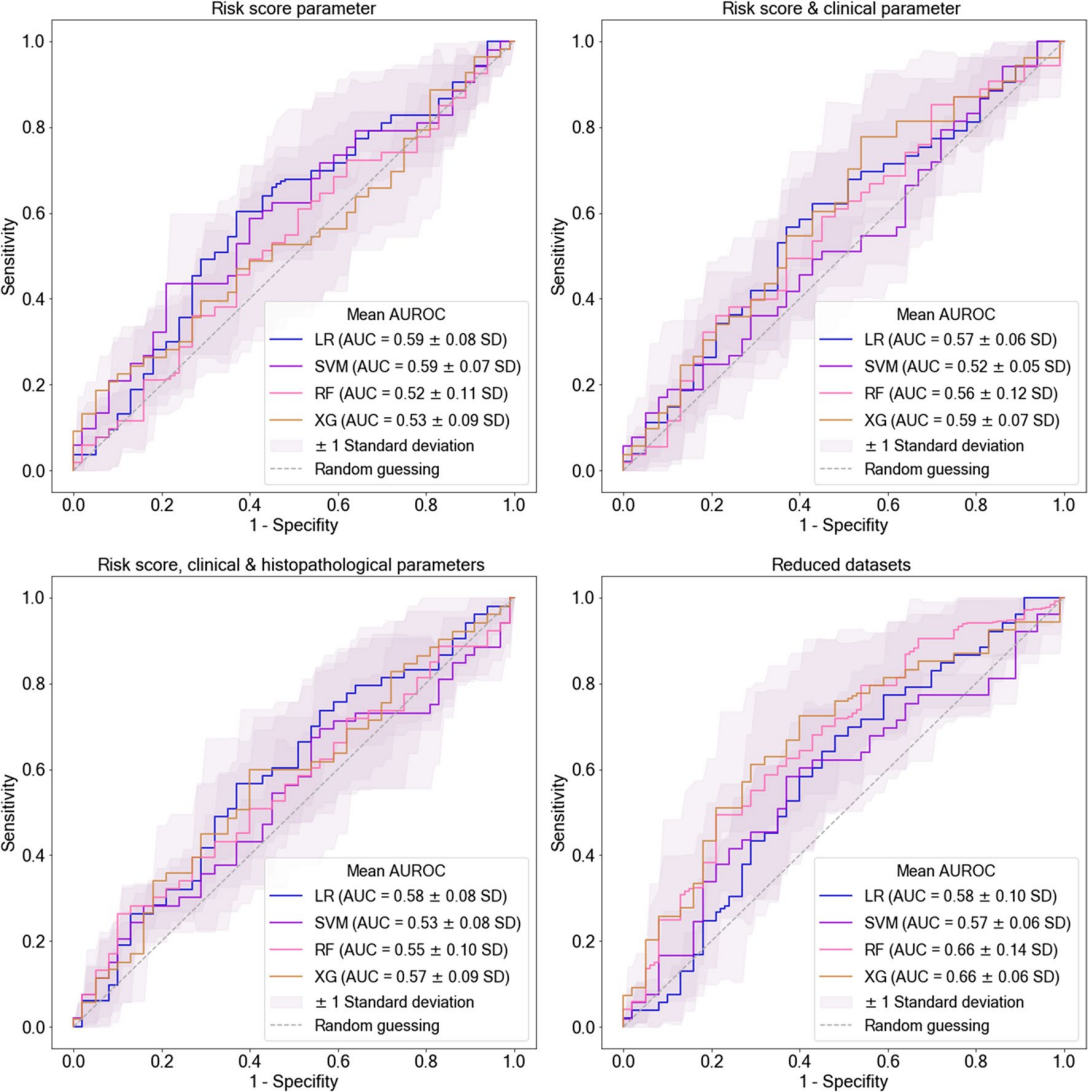
ROC curves of models trained on different datasets to predict *accelerated declining eGFR slope*. Best model predictions were observed for Random Forest (RF) and XG Boost (XG) after Machine Learning-driven feature selection (reduced datasets = selected feature datasets). Model performances after training with the other datasets did not vary. ROC curves and AUC are represented as mean of the fivefold nested cross-validation scores. *LR* logistic regression, *SVM* support vector machine, *SD* standard deviation

The performance of all models trained on the two predefined data subsets or the entire dataset to predict *accelerated declining eGFR slope* three years post-donation ranged between 0.29 and 0.41 for the *k*-fold *F*1 Score, and between 0.52 and 0.59 for the *k*-fold AUC (Table 2, Fig. 2). Machine learning models did not outperform logistic regression. No major differences in predictive performance were observed between the models.

**Table 2:** Respective model performance after predicting *accelerated declining eGFR slope* with nested cross-validation (CV, *k*-folds = 5) using different datasets

	Logistic regression	Random forest	XG boost	Support vector machine
*Dataset 1* Risk score parameters				
*k*-fold *F*1 score (SD)	0.35 (0.06)	0.35 (0.09)	0.34 (0.04)	0.41 (0.06)
*k*-fold AUC (SD)	0.59 (0.08)	0.52 (0.11)	0.53 (0.09)	0.59 (0.07)
*Dataset 2* Risk score and clinical parameters				
*k*-fold *F*1 score (SD)	0.32 (0.08)	0.37 (0.07)	0.34 (0.09)	0.29 (0.09)
*k*-fold AUC (SD)	0.57 (0.06)	0.56 (0.12)	0.59 (0.07)	0.52 (0.05)
*Dataset 3* Risk score, clinical and histopathological parameters				
*k*-fold *F*1 score (SD)	0.36 (0.01)	0.37 (0.08)	0.37 (0.08)	0.38 (0.03)
*k*-fold AUC (SD)	0.58 (0.08)	0.55 (0.10)	0.57 (0.09)	0.53 (0.08)
*Selected feature datasets* After ML-driven Feature Selection				
*k*-fold *F*1 score (SD)	0.37 (0.01)	0.44 (0.08)	0.44 (0.08)	0.35 (0.04)
*k*-fold AUC (SD)	0.58 (0.10)	0.66 (0.14)	0.66 (0.06)	0.57 (0.06)

*SD* standard deviation

Performance metrics are represented as *k*-fold area under the curve (AUC, mean of fivefold AUCs) and *k*-fold *F*1 score (mean of fivefold *F*1 scores)

Models trained with *Dataset 1* containing only parameters that count into the donor risk score for ESKD predicted the *accelerated declining eGFR slope* with a *k*-fold *F*1 score (SD) of 0.35 (0.06), 0.35 (0.09), 0.34 (0.04) and 0.41 (0.06) for logistic regression, random forests, extreme gradient boosting and *s*upport vector machines, respectively. The *k*-fold AUC (SD) was 0.59 (0.08 and 0.07) for both logistic regression and support vector machines, 0.52 (0.11) for random forests and 0.53 (0.09) for extreme gradient boosting.

We expanded *Dataset 1* by adding more clinical features of the medical history and renal cortex volumetry (= *Dataset 2*). When trained with *Dataset 2*, *k*-fold AUC of the ensemble machine learning methods—random forests and extreme gradient boosting—improved compared to when only trained with the smaller *Dataset 1* (*k*-fold AUC [SD]: 0.56 [0.12]/0.59 [0.07] random forests/extreme gradient boosting. *K*-fold *F*1 Score (SD) of random forests equally improved (0.37 [0.07]), while *F*1 Score of extreme gradient boosting remained constant. On the contrary, model performance dropped for logistic regression and support vector machines when adding more clinical features to the dataset (*k*-fold *F*1 Score [SD]: 0.32 [0.08]/0.29 [0.09]; *k*-fold AUC [SD]: 0.57 [0.06]/0.52 [0.05] logistic regression/support vector machines).

Finally, the whole dataset, including histopathological parameters of the time-zero biopsy, was used to train the models (= *Dataset 3*). Performance metrics of the models remained at a similar level to when trained with *Dataset 2*.

### Improved model predictions after feature selection

We performed machine learning-driven feature selection with sequential forward selection to find the best predictive features for model performance and to exclude redundant features. The best features were derived for each model individually from the whole dataset and were used to retrain the models.

The features found after machine learning-driven feature selection differed in type and number, and are displayed in Supplementary Fig. 2. The new datasets were reduced from 26 one-hot-encoded features of the original dataset to 8 for logistic regression and support vector machines, and to 6 one-hot-encoded features for random forests and extreme gradient boosting, respectively. The number of these best features is comparable to *Dataset 1,* which included only the ESKD risk score parameters.

When retrained on the respective selected features, random forests and extreme gradient boosting revealed the best overall performance when predicting *accelerated declining eGFR slope* (Table 2, Fig. 2)*.* Performance metrics could be raised to a *k*-fold AUC (SD) of 0.66 (0.14 random forests, 0.06 extreme gradient boosting) and a *k*-fold *F*1 score (SD) of 0.44 (0.08 random forests and *extreme gradient boosting*), respectively. On the contrary, predictive performance of logistic regression and support vector machines remained similar to the performance when trained with the whole dataset.

We compared the best-selected features between all models. Two patient attributes appeared in all four selected feature datasets: features related to smoking (the smoking history or pack years) and Banff Lesion Score *g* (Supplementary Fig. 2).

## Discussion

This study was conducted to test the ability of machine learning models to preoperatively predict relevant deterioration of excretory kidney function following kidney donation. Scientific research is mainly focused on predicting the outcome of graft function, which has been previously attempted using machine learning [9–11]. Contrary to findings of older studies, kidney donors were shown to be at increased risk of developing ESKD [3, 4]. Identifying these at-risk donors is still an unmet need in clinical practice.

We used the eGFR slope as our target for predictions. As a dynamic parameter, we consider the eGFR slope to be a better parameter for assessing donor kidney function than just eGFR at a specific time point during follow-up. Particularly for donors with borderline pre-donation eGFR, the extent of eGFR changes over time provides a more comprehensive picture of the current kidney function compared to past time-points, and reflects the approach of clinicians by putting eGFR in a temporal context.

The use of eGFR slope as a surrogate parameter to evaluate kidney function has been discussed in previous literature [35–39]. A recently published meta-analysis reported associations between treatment effects altering the GFR slope and the respective clinical endpoints targeting worsening kidney function. The authors concluded that GFR slope serves as a good surrogate parameter for evaluating kidney function in clinical trials [38], which has also been considered by regulatory agencies such as the U.S. *Food and Drug Administration* (FDA) [40] and the *European Medicines Agency* (EMA) [41].

A normal decline in kidney function is approximately 1 mL/min/1.73 m^2^/year [18]. The median eGFR slope of our donor collective was − 0.33 mL/min/1.73 m^2^/year, which is consistent with previous findings reporting the measured GFR slope of donors to be around − 0.4 mL/min/1.73 m^2^/year [2, 42]. Based on these findings, we defined a relevant eGFR slope at − 1 mL/min/1.73 m^2^/year in the third follow-up year. This resulted in an unbalanced dataset with 185 donors in the *average eGFR slope* cohort and 53 donors in the *accelerated declining eGFR slope* cohort.

Neither the ESKD risk score nor the descriptive statistics of the other pre-donation donor features used for model training effectively discriminated the donor cohort with the *accelerated declining eGFR slope*. Therefore, we employed machine learning to effectively identify this donor cohort. Three machine learning models (random forests, extreme gradient boosting, support vector machines) and logistic regression as the state-of-the-art model were utilized to predict *accelerated declining eGFR slope* of our donor cohort. Overall, no model sufficiently predicted the outcome. Neither of the models exceeded an AUC of 0.7 or an *F*1 score of 0.5.

Also, Jeon et al. [12] reported mediocre performance with machine learning in predicting the percentage of renal adaptation (6–12 months post-donation eGFR/pre-donation eGFR, cut-off: 65% of pre-donation eGFR after donation) of kidney donors after training with preoperatively assessed donor features. The authors reported an AUC of 0.63, which is similar to our results. They additionally trained the machine learning model to predict the absolute median eGFR of the second half of the first follow-up year (cut-off: 60 mL/min/1.73 m^2^). Here, clearly improved model performance with an AUC of 0.85 was observed. However, we consider predicting excretory kidney function decline to be superior to predicting GFR alone, as discussed above.

Despite the low predictive performance of the machine learning models, there are some observed trends of the distinct model performances when trained on different data subsets. The first data subset we used for model training included patient characteristics for calculating the ESKD risk score for kidney donors. The risk score was first introduced in 2016 by Grams et al. [7] after observing more than 4,000,000 individuals who were formally eligible for kidney donation, for 4–16 years. In our transplant center, we use this risk score to screen for potential donor candidates and to exclude donors at risk. Our interest was to find out whether these well-established parameters are sufficient to predict *accelerated declining eGFR slope* with machine learning.

The calculated 15-year and lifetime ESKD risk score for our donor cohort was below 1% for both eGFR-slope cohorts. Interestingly, a statistically significant difference was noted for the 15-year ESKD risk score. However, the differences in the absolute values were marginal. The calculated risk scores themselves were not included in model training. Likewise, we did not consider non-insulin dependent diabetes and race for model training due to one-dimensionality in our patient cohort.

The best performance using the risk score dataset was noted for support vector machines, which are known to be efficient with small datasets [43]. However, differences in model performance compared to the other models were marginal. In our study, machine learning models failed to adequately predict *accelerated declining eGFR slope* after being trained on previously evaluated patient characteristics for ESKD risk-prediction.

Subsequently, we integrated more features into the dataset and expected improved predictions related to the greater amount of information. We included additional donor details such as body weight, height, pack years, or renal cortex volumetry from CT scans (*Dataset 2*). For the entire dataset (*Dataset 3*), results of the time-zero biopsy, including Banff Lesion Scores, were added. Even though the histology of living donor kidneys is not available in pre-donation screening, results of the time-zero biopsy might affect the remaining renal outcome of living kidney donors.

Including more parameters led to slightly better results for random forests and extreme gradient boosting but worsened the predictions for support vector machines. Logistic regression showed consistent performance across the different data subsets. Barah et al. [44] also reported a slight improvement in model performance for predicting kidney discard with machine learning after adding parameters from the graft biopsy. Nevertheless, expanding the dataset with predefined features did not improve predicting donors with an *accelerated declining eGFR slope*.

Finally, we applied machine learning-driven feature selection to the whole dataset. We used model agnostic sequential feature selection in a forward approach by sequentially adding the most informative features to enhance model performance in *k*-fold cross-validation [20]. After sequential forward selection, each model exhibited a different subset of best predictive features. Eight and six best predictive features were found for logistic regression/support vector machines and extreme gradient boosting/random forests, respectively. We then retrained each model with the respective selected features. A clear improvement in prediction was observed for extreme gradient boosting and random forests. Both ensemble methods revealed a *k*-fold AUC of 0.66 and a *k*-fold *F*1 score of 0.44, and outperformed logistic regression and support vector machines which did not show improved predictive performance. These findings are consistent with previous machine learning studies in kidney transplantation: Feature selection improved predictive performance [9], and random forests or extreme gradient boosting outperformed logistic regression [9, 10, 44].

The best predictive features that appeared in all four models after sequential forward selection were the features related to smoking, namely smoking history or pack years, and the Banff Lesion Score *g* (glomerulitis). Smoking as a cardiovascular risk factor is widely known to enhance the incidence of developing chronic kidney disease [45]. Therefore, it is not surprising that all four models use features related to smoking to improve predictions for *accelerated declining eGFR slope*.

The Banff classification is designed for allograft pathologies [15]. Nevertheless, pathologies in the time-zero biopsy provide insights about the donor’s remaining kidney. The Banff *g* lesion score classifies the proportion of microvascular inflammation within glomeruli which may be linked to antibody-mediated graft rejection or to recurrent or de novo glomerulonephritis [15]. Previous studies reported that glomerulitis was associated with allograft pathologies or graft failure [46–50]. The conclusive determination of whether the reasons for glomerulitis may be recipient-associated, such as humoral rejection or recurrence of an underlying condition, is hindered by inconsistent documentation regarding the timing of biopsy acquisition in relation to reperfusion. Whether the presence of glomerulitis in the time-zero biopsy of the graft allows a conclusion to be drawn about the outcome of the remaining kidney function of living kidney donors needs to be investigated in further studies.

From a data science perspective, we faced a few hurdles that accounted for the moderate model performances. We trained our models on a small dataset that was unbalanced and consisted of missing values. There is a widespread belief that artificial intelligence is designed to only recognize patterns in large amounts of data. However, small datasets are common in the medical field. Althnian et al. [51] empirically investigated the influence of data size on the performance of machine learning models using datasets from the medical domain. They found that it is not the data size itself that affects the predictive ability, but rather how closely the data reflect the general distribution of a patient cohort. These findings are consistent with the results of our study: Not including more data but identifying the predictive features and retraining the models without redundant features improved the predictions.

The limitations of our study are that we used the eGFR values instead of measured GFR to calculate the eGFR slope. Our dataset consisted of missing values, mainly due to incomplete documentation of the histopathological parameters. There are no gold standards in data science for the allowed number of missing values in a dataset, which, thus, remains a field of empirical testing. We did not include all parameters that define the Banff classification due to one-dimensionality. Our dataset stemmed from one transplantation center. The performance of the machine learning models was evaluated by *k*-fold cross-validation which allows to investigate the ability of the models to generalize the information. To further test the predictive performance and generalizability of the models, an external test set is required for validation.

## Conclusion

Our aim was to predict *accelerated declining eGFR slope* of living kidney donors using machine learning. Training the models with distinct predefined data subsets did not produce satisfactory predictions for any model. However, the predictive performance of the random forests and extreme gradient boosting improved and outperformed logistic regression after training with only important features after machine learning-driven feature selection. Future studies need to be conducted with extended data size to evaluate whether machine learning can sufficiently predict the eGFR slope to identify donors at risk for declining kidney function.

## Supplementary Information

Below is the link to the electronic supplementary material.

Supplementary file1 (DOCX 835 KB)

## Data Availability

The dataset generated during the current study is available from the corresponding author on reasonable request.

## Abbreviations

ACR: Urine albumin creatinine ratio
AI: Artificial intelligence
AUC: Area under the curve
BMI: Body mass index
CKD-EPI: Chronic Kidney Disease Epidemiology Collaboration
CV: Cross-validation
CT: Computed tomography
eGFR: Estimated glomerular filtration rate
ESKD: End-stage kidney disease
HARP: Hand-assisted retroperitoneoscopic donor nephrectomy
IQR: Interquartile range
LR: Logistic regression
ML: Machine learning
RF: Random forest
SD: Standard deviation
SFS: Sequential forward selection
SVM: Support vector machine
XG: Extreme gradient (XG) boosting
