# Supplementary material for: First experiences with machine learning predictions of accelerated declining eGFR slope of living kidney donors 3 years after donation
Source: J Nephrol. 2024 Jun 5;37(6):1631–42. doi: 10.1007/s40620-024-01967-y (PMC11473598; doi:10.1007/s40620-024-01967-y)
Supplement: Supplementary file 1 — Supplementary file1 (DOCX 835 KB) [file 40620_2024_1967_MOESM1_ESM.docx]

# Supplementary Literature

31. Waskom, M.L., *Seaborn: statistical data visualization.* Journal of Open Source Software, 2021. **6**(20): p. 3021. DOI: 10.21105/joss.03021

32. Bilogur, A., *Missingno: a missing data visualization suite.* Journal of Open Source Software, 2018. **3**(22): p. 547. DOI: 10.21105/joss.00547

33. Virtanen, P., et al., *SciPy 1.0: fundamental algorithms for scientific computing in Python.* Nature Methods, 2020. **17**(3): p. 261-272. DOI: 10.1038/s41592-019-0686-2

34. Team, R. *RStudio: Integrated Development for R. RStudio*. 2020; Available from: <http://www.rstudio.com/>.

35. Grams, M.E., et al., *Evaluating Glomerular Filtration Rate Slope as a Surrogate End Point for ESKD in Clinical Trials: An Individual Participant Meta-Analysis of Observational Data.* J Am Soc Nephrol, 2019. **30**(9): p. 1746-1755. DOI: 10.1681/asn.2019010008

36. Greene, T., et al., *Performance of GFR Slope as a Surrogate End Point for Kidney Disease Progression in Clinical Trials: A Statistical Simulation.* J Am Soc Nephrol, 2019. **30**(9): p. 1756-1769. DOI: 10.1681/asn.2019010009

37. Inker, L.A. and J. Chaudhari, *GFR slope as a surrogate endpoint for CKD progression in clinical trials.* Curr Opin Nephrol Hypertens, 2020. **29**(6): p. 581-590. DOI: 10.1097/mnh.0000000000000647

38. Inker, L.A., et al., *A meta-analysis of GFR slope as a surrogate endpoint for kidney failure.* Nature Medicine, 2023. **29**(7): p. 1867-1876. DOI: 10.1038/s41591-023-02418-0

39. Inker, L.A., et al., *GFR Slope as a Surrogate End Point for Kidney Disease Progression in Clinical Trials: A Meta-Analysis of Treatment Effects of Randomized Controlled Trials.* J Am Soc Nephrol, 2019. **30**(9): p. 1735-1745. DOI: 10.1681/asn.2019010007

40. Thompson, A., K. Smith, and J. Lawrence, *Change in Estimated GFR and Albuminuria as End Points in Clinical Trials: A Viewpoint From the FDA.* Am J Kidney Dis, 2020. **75**(1): p. 4-5. DOI: 10.1053/j.ajkd.2019.08.007

41. Holtkamp, F., et al., *Change in Albuminuria and Estimated GFR as End Points for Clinical Trials in Early Stages of CKD: A Perspective From European Regulators.* Am J Kidney Dis, 2020. **75**(1): p. 6-8. DOI: 10.1053/j.ajkd.2019.07.019

42. Berglund, D.M., et al., *Measured Glomerular Filtration Rate After Kidney Donation: No Evidence of Accelerated Decay.* Transplantation, 2018. **102**(10): p. 1756-1761. DOI: 10.1097/tp.0000000000002215

43. Cervantes, J., et al., *A comprehensive survey on support vector machine classification: Applications, challenges and trends.* Neurocomputing, 2020. **408**: p. 189-215. DOI: 10.1016/j.neucom.2019.10.118

44. Barah, M. and S. Mehrotra, *Predicting Kidney Discard Using Machine Learning.* Transplantation, 2021. **105**(9): p. 2054-2071. DOI: 10.1097/tp.0000000000003620

45. Kalantar-Zadeh, K., et al., *Chronic kidney disease.* Lancet, 2021. **398**(10302): p. 786-802. DOI: 10.1016/s0140-6736(21)00519-5

46. Gonzales, M.M., et al., *Predicting Individual Renal Allograft Outcomes Using Risk Models with 1-Year Surveillance Biopsy and Alloantibody Data.* J Am Soc Nephrol, 2016. **27**(10): p. 3165-3174. DOI: 10.1681/asn.2015070811

47. Hara, S., et al., *Allograft glomerulitis: histologic characteristics to detect chronic humoral rejection.* Transplant Proc, 2005. **37**(2): p. 714-6. DOI: 10.1016/j.transproceed.2004.11.077

48. Messias, N.C., et al., *Cohort study of the prognostic significance of acute transplant glomerulitis in acutely rejecting renal allografts.* Transplantation, 2001. **72**(4): p. 655-60. DOI: 10.1097/00007890-200108270-00016

49. Nabokow, A., et al., *Long-term kidney allograft survival in patients with transplant glomerulitis.* Transplantation, 2015. **99**(2): p. 331-9. DOI: 10.1097/tp.0000000000000606

50. Whigham, M., et al., *Impact of Glomerulitis on Long-term Outcomes After Kidney Transplantation.* Transplant Direct, 2022. **8**(10): p. e1381. DOI: 10.1097/txd.0000000000001381

51. Althnian, A., et al., *Impact of Dataset Size on Classification Performance: An Empirical Evaluation in the Medical Domain.* Applied Sciences, 2021. **11**(2): p. 796. DOI: 10.3390/app11020796

# Supplementary Table

Supplementary Table 1. Hyperparameter search space for nested cross-validation.

|  | Hyperparameter search space |
| --- | --- |
| Logistic Regression | penalty = l2  solver = [newton-cg, lbfgs, liblinear, sag, saga]  max_iter = range (100, 1000, 10))  tol = linspace (1e-6, 1e-2, 10)  C = linspace (0.001, 10, 20)  class_weight = [{0:x, 1:1.0-x} for x in linspace (0.05,0.95,50)]} |
| Support Vector machine | C = linspace (0.01, 10,10)  kernel = [linear, poly, rbf, sigmoid]  gamma = linspace (0.001, 10,10)  max_iter = range (100, 1000, 10)  tol = linspace (1e-6, 1e-2, 10)  class_weight = [{0:x, 1:1.0-x} for x in linspace (0.05,0.5,50)]} |
| Random Forest | n_estimators = range (20, 500, 50)  max_depth = range (5, 25, 2)), None  min_samples_leaf = [1,2,3]  min_samples_split = [2, 3, 4]  max_features = [auto, sqrt],  bootstrap = [True, False]  class_weight = [{0:x, 1:1.0-x} for x in linspace (0.05,0.95,50)]} |
| XG Boost | n_estimators = range (10, 110, 5))  max_depth = range (3, 10, 1)), None  min_child_weight = range (1, 5, 1)  learning_rate = linspace (0.01,0.3,10)  colsample_bytree = [0.5, 0.6, 0.7, 0.8]  subsample = [0.5, 0.6, 0.7, 0.8]  reg_lambda = [0.1, 0.5, 1]  gamma = [ 0.0, 0.1, 0.2, 0.4, 1.0]  scale_pos_weight = linspace(2,10,20) |

# Supplementary Figures


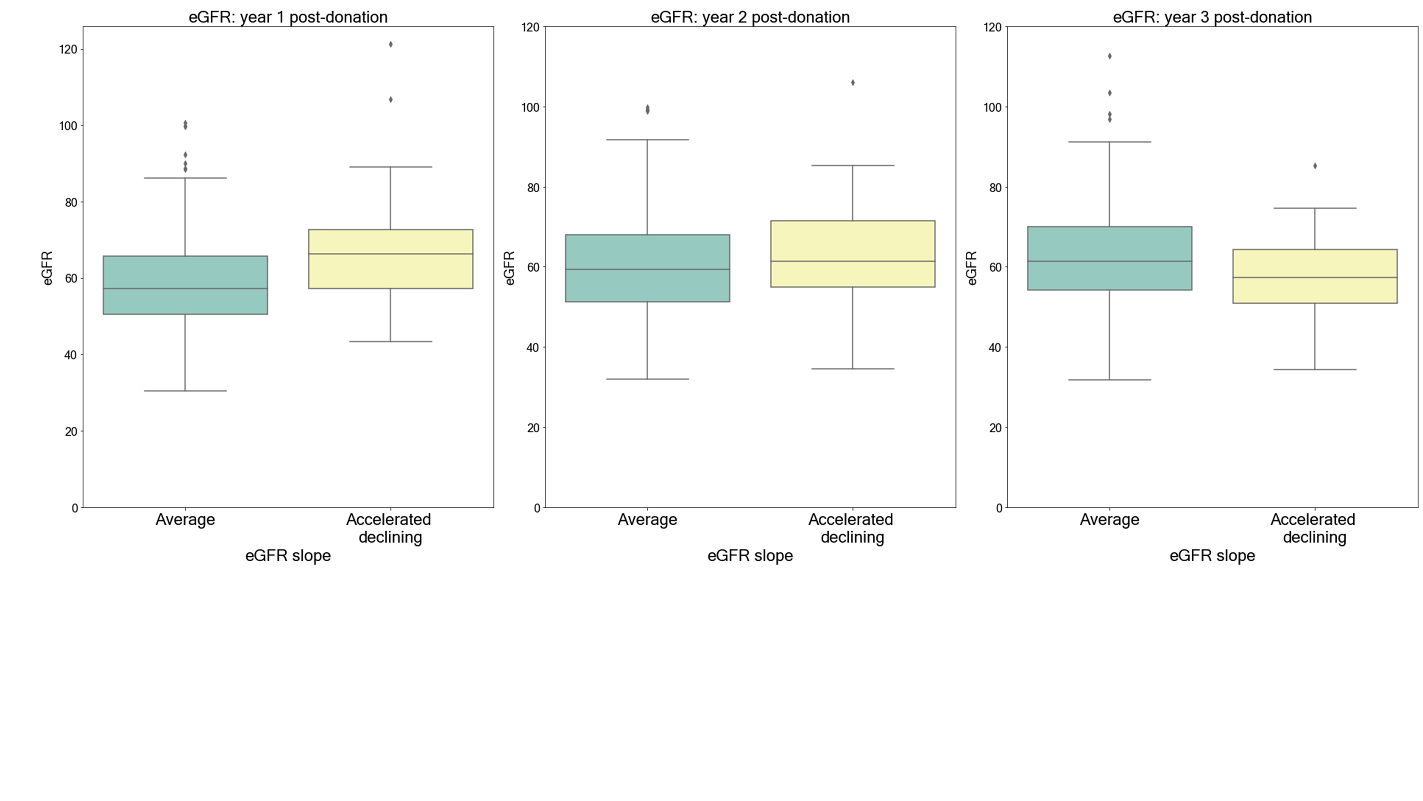


Supplementary Figure 1. eGFR trajectories of kidney donors over the course of follow-up after living kidney donation stratified into eGFR slope cohort-groups of the third follow-up year. The eGFR (estimated glomerular filtration rate, ml/min/1.73 m²) is depicted for the first, second, and third follow-up years after living kidney donation (N = 238). Data is stratified according to eGFR slope groups (*average* [n = 185] vs. *accelerated* *declining* [n = 53], cut-off = -1 ml/min/1.73 m²/year) calculated specifically for the third follow-up year. Statistical analysis reveals a significant difference between the two cohorts in both the one- and three-year follow-up periods (p < 0.001, p = 0.006, respectively).


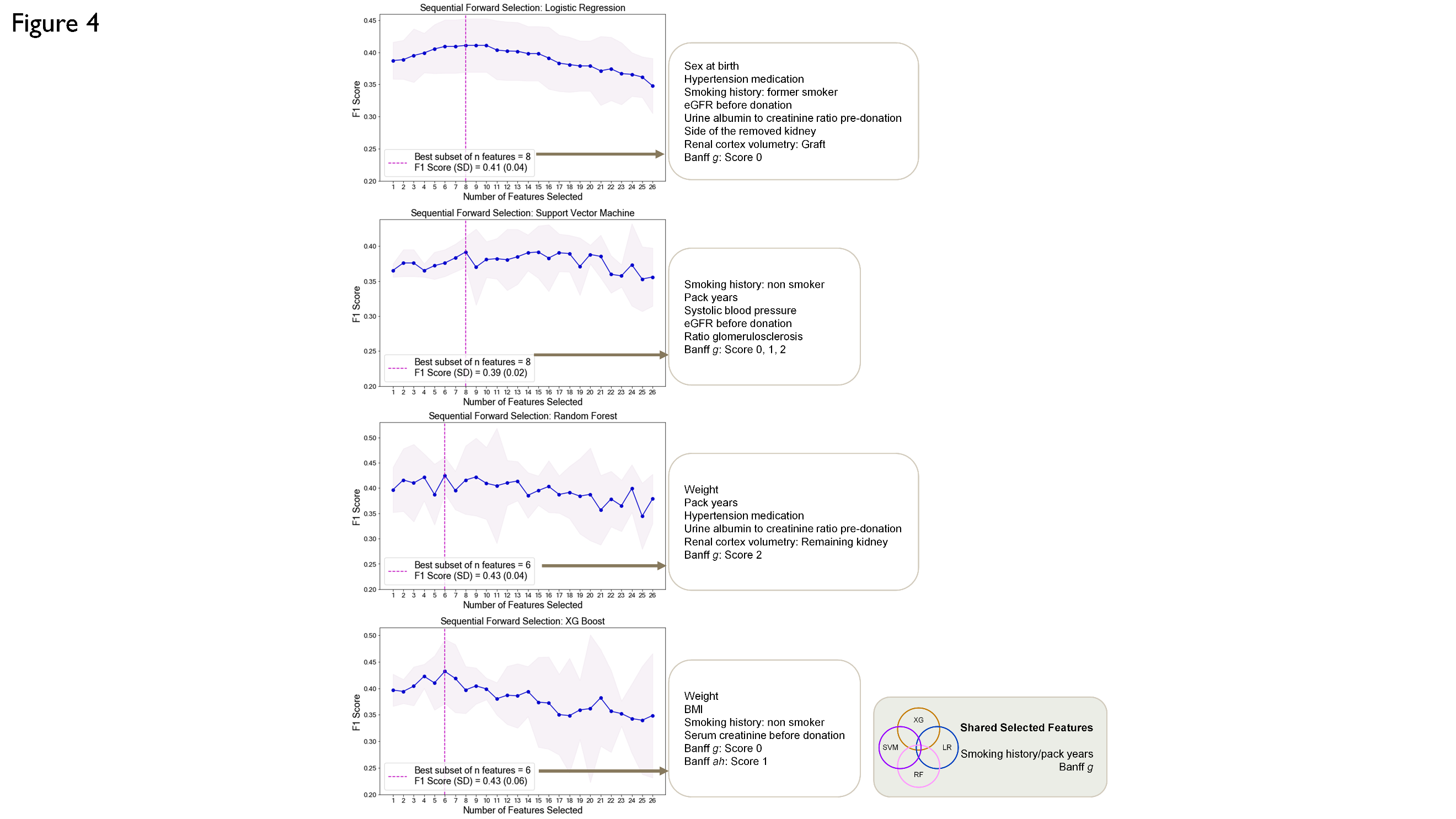


Supplementary Figure 2. Machine Learning-driven feature selection with model-agnostic sequential forward selection (SFS). The best features to improve model performance (F1 score) were derived for each model individually from the entire dataset. The features found after feature selection differed in type and number for each model. Shared selected features after SFS among all models were features related to smoking (smoking history and pack years) and Banff Lesion Score for glomerulitis (g). Shaded regions represent ± 1 standard deviation (SD).
